# A novel fusion algorithm for benign-malignant lung nodule classification on CT images

**DOI:** 10.1186/s12890-023-02708-w

**Published:** 2023-11-28

**Authors:** Ling Ma, Chuangye Wan, Kexin Hao, Annan Cai, Lizhi Liu

**Affiliations:** 1 College of Software, Nankai University, Tianjin, 300350 China; 2 Department of Radiology, Sun Yat-Sen University Cancer Center, Guangzhou, 510060 Guangdong China

**Keywords:** Lung cancer, Lung nodule classification, Information fusion, Deep convolutional neural network, Graph convolutional network, Computed tomography (CT)

## Abstract

The accurate recognition of malignant lung nodules on CT images is critical in lung cancer screening, which can offer patients the best chance of cure and significant reductions in mortality from lung cancer. Convolutional Neural Network (CNN) has been proven as a powerful method in medical image analysis. Radiomics which is believed to be of interest based on expert opinion can describe high-throughput extraction from CT images. Graph Convolutional Network explores the global context and makes the inference on both graph node features and relational structures. In this paper, we propose a novel fusion algorithm, RGD, for benign-malignant lung nodule classification by incorporating Radiomics study and Graph learning into the multiple Deep CNNs to form a more complete and distinctive feature representation, and ensemble the predictions for robust decision-making. The proposed method was conducted on the publicly available LIDC-IDRI dataset in a 10-fold cross-validation experiment and it obtained an average accuracy of 93.25%, a sensitivity of 89.22%, a specificity of 95.82%, precision of 92.46%, F1 Score of 0.9114 and AUC of 0.9629. Experimental results illustrate that the RGD model achieves superior performance compared with the state-of-the-art methods. Moreover, the effectiveness of the fusion strategy has been confirmed by extensive ablation studies. In the future, the proposed model which performs well on the pulmonary nodule classification on CT images will be applied to increase confidence in the clinical diagnosis of lung cancer.

## Introduction

Cancer is one of the most deadly diseases and lung cancer is the most common and among the deadliest cancers in the world [1]. Recent studies show that the 10-year relative survival rate can increase by 20% through annual screening with low-dose computed tomography (CT) in early stages to capture the cancer heterogeneity in a non-invasive way [2–4] and most lung cancers arise from small malignant nodules [3, 5]. However, malignant nodule recognition by radiologists reading every CT scan is extremely time-consuming and subjective. Therefore, many computer-aided diagnosis (CAD) systems being powerful tools to aid radiologists in identifying malignant lung nodules have been proposed in recent years [6]. Most current CAD systems for lung nodule classification can be divided into two respects, one is handcraft-based CAD systems, and the other is deep learning-based CAD systems.

The handcraft-based CAD systems used the intensity, texture, and shape features, like local binary patterns [7], wavelet features [8], histogram of oriented gradient (HOG) [9], gray level co-occurrence matrix (GLCM) [10], 3D surface feature [11], selected the distinguishing features [12–14] and then trained a designed classifier, such as the support vector machine (SVM) [15], Bayesian [16], and random forest [17], or combined classifier [18], for pulmonary nodule classification.

The deep learning-based CAD systems used convolutional neural networks (CNNs) for lung nodule classification from CT images [19, 20]. Many deep learning-based CAD systems have achieved good performance for lung nodule classification. However, benign and malignant nodules sometimes look similar and hard to distinguish from one another, and most deep learning-based CAD systems use the CNN models which include a set of convolutional and pooling layers that can be operated on the data with a Euclidean structure. Hence, exploring and fusing the non-Euclidean characteristic becomes an attractive and reasonable choice to improve the classification results. Graph convolutional networks (GCNs) have a great expressive power to learn non-Euclidean representations and have demonstrated superior performance in the classification task [21].

In this paper, we propose a fusion algorithm, RGD, combining the Radiomics, and GCN features into the Deep CNNs to form a more complete feature representation for benign-malignant lung nodule classification on CT images. First, we calculate the radiomics features to describe high-throughput extraction from the lung nodules from CT images, because pathological studies have demonstrated that there is increased heterogeneity within malignant lung nodules, which is not appreciable in radiological studies by the naked eye but can be quantified with radiomics [22]. Second, we adopt several different CNN models to extract the self-learned features. Next, these deep features are fed into a graph convolutional network to model the relations among features and furnish more promising insights underlying the features in non-Euclidean space. Finally, ensemble learning fuses the informative knowledge to achieve knowledge discovery and better predictive performance via voting schemes in an adaptive way. To the best of our knowledge, this is the first work fusing the radiomics, deep CNN features, and graph features representation for lung nodule classification on CT images. Compared with the existing simple concatenation or fully connected fusion methods, our proposed RGD algorithm can effectively separate malignant from benign nodules by fusing several CNN features in graph structure to mine the discriminating features and eliminate the redundant representations.

## Related work

In recent years, deep learning methods have gradually been applied and designed for lung nodule classification.

Some methods designed a special CNN architecture for the lung nodule classification by involving the 2D and 3D space information [23], multi-scale [24, 25], multi-view [26, 27], multi-crop [28], local-global information [29], dilated convolutions [30], attention mechanism [31], manifold representation [32], lung nodule shape and margin feature analysis [33].

Some methods fused multiple models to improve the classification ability. Dey et al. proposed a fused model for the problem of diagnostic classification between benign and malignant lung nodules in CT images, including a basic 3D CNN, a novel multi-output network, a 3D DenseNet, and an augmented 3D DenseNet with multi-outputs [34]. Zhao et al. constructed a hybrid CNN of LeNet and AlexNet by combining the layer settings of LeNet and the parameter settings of AlexNet for pulmonary nodule classification [35]. Zhao et al. integrated different CNNs and adopted transfer learning to utilize deep convolutional neural networks for lung nodule classification [36]. Wang et al. proposed an adaptive-boost deep learning method by composing a series of multi-layer perceptions (MLPs) to obtain a strong classifier [37]. Onishi et al. used the Wasserstein GAN to generate the multiplanar images of the pulmonary nodule and performed three DCNNs on these generated nodule images for pulmonary nodule classification in CT images [38]. Xu et al. proposed a method called MSCS-DeepLN to evaluate lung nodule malignancy. They trained and combined three CNN models with multi-scale input cropped from CT images for learning the multi-level contextual features and preserving diversity [39]. Jiang et al. used attentive and ensemble 3D dual path networks (DPNs) to improve the representation ability for pulmonary nodules classification [40]. Jiang et al. used neural architecture search to automatically search 3D network architectures and fused the outputs of *n* different neural architectures to get the final prediction of pulmonary nodules [41]. Liu et al. used the combinations of multiple CNNs and multiple traditional machine learning models, like k-nearest-neighbor, logistic regression, and SVM, based on handcrafted features in terms of morphology, density, curvature, and margin gradient, for predicting the likelihood of malignancy of pulmonary nodules from the CT scans [42].

Some methods fused multiple tasks to improve the performance of pulmonary nodule classification. Hussein et al. proposed a 3D CNN-based multi-task learning for nodule classification. They acquired another task-dependent feature representation for six high-level nodule attributes for improving the benign-malignant nodule classification accuracy [43]. Dai et al. proposed a new method named attribute-lung-nodule classification to combine the two classification tasks, pulmonary nodule benign-malignant classification, and pulmonary nodule image attributes classification, into a deep learning network to improve the accuracy of pulmonary nodule classification [44]. Khosravan et al. proposed a 3D deep multi-task CNN to tackle these two problems, false positive nodule reduction, and nodule segmentation jointly to improve the computer-aided diagnosis systems’ performance [45].

Some methods fused multiple features to improve the benign-malignant nodule classification accuracy. Chen et al. combined the general low-level Haarlike and HOG features, the description of nine semantic features, and the heterogeneous computational features derived from the deep learning models to provide richer quantitative assessments of nodules for better support of the diagnostic decision and management [46]. Buty et al. used both appearance distinctions described by deep convolutional neural networks and 3D surface variations modeled by spherical harmonics to character the lung nodules [47]. Wang et al. fused the hand-crafted and deep features to train the cost-sensitive random forest for classifying the lung nodules in chest X-ray images [48]. Zhu et al. constructed features by concatenating the learned deep 3D DPN features, nodule size, and raw 3D cropped nodule pixels and employed a gradient boosting machine for pulmonary nodule classification [49]. Xie et al. proposed an algorithm that fused the texture features by employing a GLCM-based texture descriptor and shape features by using a Fourier shape descriptor to characterize the heterogeneity of nodules and deep convolutional neural network model-learned information at the decision level for lung nodule classification [50]. Li et al. proposed a fusion algorithm that combined the twenty-nine handcrafted features including nine intensity features, eight geometric features, and twelve texture features based on GLCM, and the CNN features from AlexNet, VGG-16, and Multi-crop Net. Then the combined features were used as the input for the SVM coupled with the sequential forward feature selection method to select the optimal feature subset and construct the classifiers [51].

Although these fused models can capture semantic and syntactic information in local descriptors well, they combine the multiple information in a simple and direct strategy and might ignore underlying complex relationships among these different types of information. In contrast, our proposed method fusing several CNN features in graph structure can capture the non-Euclidean representation and relational features for distinguishing malignant and benign lung nodules.

In this work, we propose a fusion algorithm, named RGD, for benign-malignant lung nodule classification on CT images by fusing the Radiomics, GCN features, and Deep CNNs features. Radiomics is the use of quantitative imaging features extracted from medical images to characterize tumor pathology or heterogeneity and radiomics features have successfully elucidated subtle relationships between image characteristics and disease status [52]. The CNN models can highly exploit the stationarity and compositionality properties and have demonstrated their power in identifying malignant lung nodules from CT data [53]. In addition, the combination of the radiomics and CNN features has been proven to be an efficient route [54]. Graph neural networks have rich relational structures and can preserve global structure information [55], and it can enable our model to explore the existing and potential relationships between neighboring features to learn discriminative features. Therefore, the proposed RGD, which fuses the Radiomic, GCN, and multiple Deep CNN features, is suitable for the feature representation. The RGD will be a powerful artificial intelligence aid for identifying malignant lung nodules in clinical practice and effectively detecting early lung cancer and then reducing mortality in lung cancer patients in the future.

## Method

**Fig. 1:**
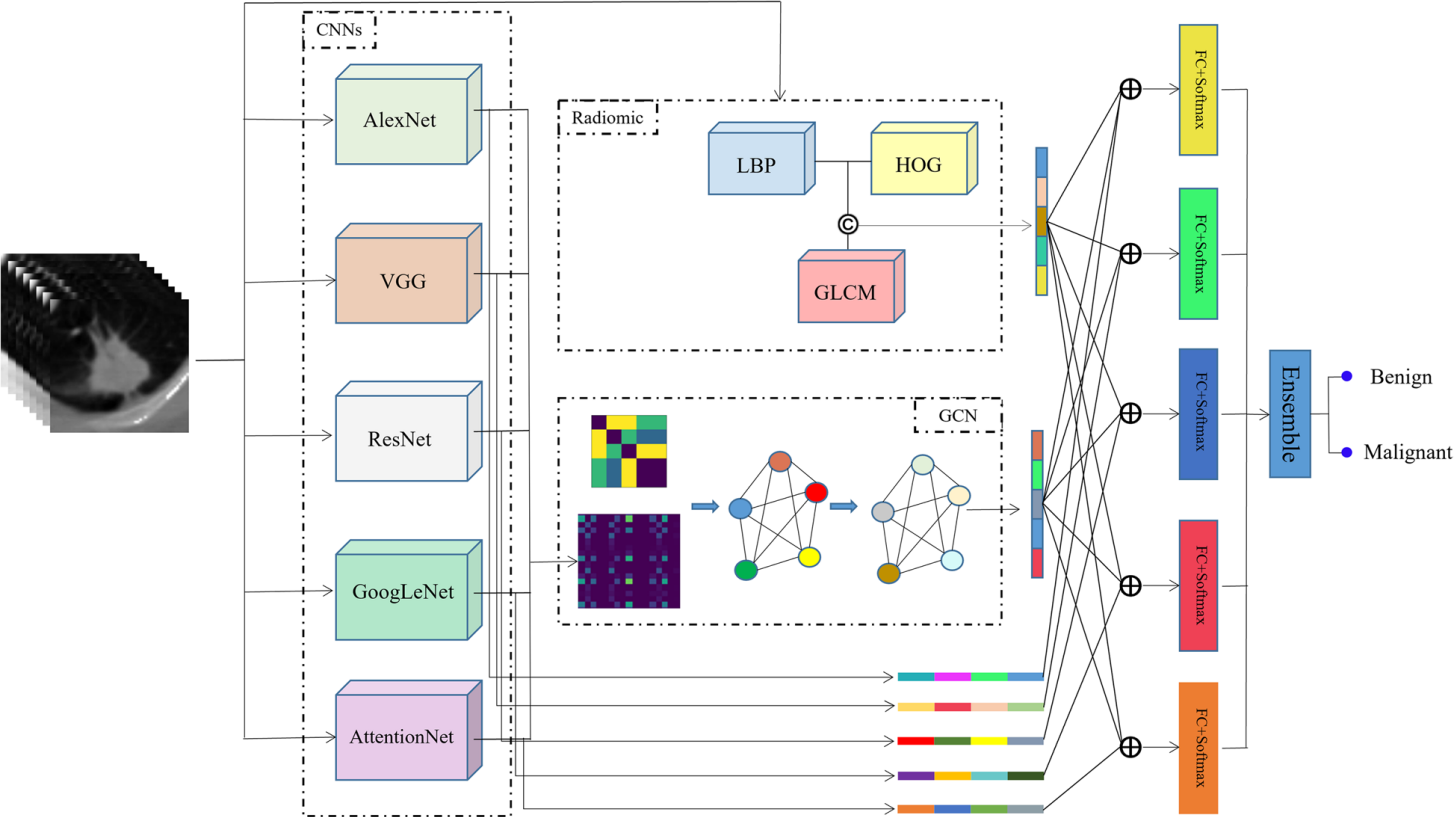
The flowchart of our RGD model

We propose a fusion algorithm for benign-malignant lung nodule classification on CT images as depicted in Fig. 1. As shown in Fig. 1, the proposed model (RGD) fuses the Radiomics, and GCN features into the Deep CNNs to form a more complete feature representation. Specifically, radiomics feature extraction is performed on CT images to describe the texture appearance. At the same time, the five different CNN models are separately trained to capture the complementary features from the CT images, then the GCN takes the CNN features as the input features and outputs the aggregated optimal features. Finally, the radiomics features and the GCN features are combined with the highest level CNN representation learned at the output layer of five 3D CNNs respectively to generate decision scores. Those decision scores from the five CNN models are ensemble for enhancing the classification performance.

### Radiomics features extraction

Perinodular and intranodular radiomics features corresponding to texture information that are believed to be of interest based on expert opinion are extracted from CT images. We involve local binary pattern (LBP), the histogram of oriented gradient (HOG), and gray level co-occurrence matrix (GLCM) for radiomics feature extraction, because of their advantages of the acquirement of more detailed information and the invariances to monotonic gray-level changes and geometric orientations.

LBP is an effective descriptor that codes the gray levels of an image by comparing the central pixel with its neighbors, and the result is counted as a binary number converted to a decimal number that substitutes the central pixel value. In our work, we use the volume LBP for modeling the texture of lung nodules in 3D CT scans. Given a central pixel in the slice, a pattern number is computed by comparing its value with those of its neighborhoods

1 $$\begin{aligned} 3DLBP_{L,P,R}=\sum \limits _{q=0}^{3P+1}{v_{q}2^{q}}, \end{aligned}$$

where *L*, *P*, and *R* mean the interval between the previous and posterior neighboring scans, the number and radius of neighbors around the central pixel in one scan, respectively. *v* is joint distribution in a local neighborhood of a series of CT scans and can be represented by

2 $$\begin{aligned} V{} & {} = v\left( \begin{array}{llll} B(g_{{s_{c}-L},c}-g_{s_{c},c}),&{}B(g_{{s_{c}-L},0}-g_{s_{c},c}),&{} \cdots ,\\ B(g_{{s_{c}-L},P-1}-g_{s_{c},c}),&{}B(g_{{s_{c}},0}-g_{s_{c},c}), &{}\cdots ,\\ B(g_{{s_{c}},P-1}-g_{s_{c},c}),&{}B(g_{{s_{c}+L},0}-g_{s_{c},c}), &{}\cdots , \\ B(g_{{s_{c}+L},P-1}-g_{s_{c},c}), &{}B(g_{{s_{c}+L},c}-g_{s_{c},c}) \end{array}\right)\nonumber \\{} & {} = v(v_0,v_1,v_2, \cdots , v_{3P+1}), B(x)= \left\{ \begin{array}{ll} 1,&{} x \ge 0 \\ 0,&{} x < 0 \end{array},\right. \end{aligned}$$

where $g_{s_{c},c}$ is the gray value of the central pixel in the slice *s* and $g_{s,p}$ is a circularly symmetric neighbor set, corresponding to the gray value of the *p* pixel on a circle of radius *R* from the previous and posterior neighboring slices. In our method, we set the *L*, *P*, and *R* as one, four, and one, respectively. According to Eq. 1, we sample and threshold neighboring points to get a binary code, and then calculate its distribution. Finally, the histograms are normalized for forming the 3D LBP features.

HOG feature vector which has a good description of the lung nodules’ appearance and involves the spatial information for the voxel’s neighbors in the features, is calculated for distinguishing between benign and malignant nodules. Each voxel within a cell is applied the filter mask $\left[ -1, 0, 1 \right]$ to its neighboring voxels in all three dimensions for calculating the gradient vector *G*, whose magnitude is obtained using azimuth $\theta$.

3 $$\begin{aligned} (\theta (x,y),\theta (z,xy))=\left(\tan ^{-1}\frac{G_y}{G_x}, \tan ^{-1}\frac{G_z}{\sqrt{{G_x}^2+{G_y}^2}}\right), \end{aligned}$$

where $\theta (x, y)$ and $\theta (z, xy)$ are the angles of the 3D gradient when projected to *XY*-plane and the angle between the gradient and z-axis, $G_x$, $G_y$, and $G_z$ are the gradients along the *X*, *Y* and *Z* directions, respectively. Then these histograms are normalized to obtain the 3D HOG features.

GLCM can reflect the second-order conditional probability distribution by counting the frequency of the pair of voxels with specific directions and distances. To generate the 3D GLCM features, we sample along the direction linking the center voxel with its nearest 26 neighbors and consider 13 (half of 26) directions because of the redundant information from the opposite directions. The distances between pixel pairs along the 13 directions in the z-axis are chosen as 1, 2, 3, and 4, and the distances along four directions on the *XY*-plane are 1, 2, 4, and 8. The co-occurrence matrix can be defined by

4 $$\begin{aligned} M_{ij}(\theta ,d)=\sum \limits _{p\in D} \left\{ \begin{array}{ll} 1 &{} {if} \quad V(p)=i \, \& \, V(p+d(\cos {\theta },\sin {\theta }))=j \\ 0 &{} otherwise \end{array}\right. , \end{aligned}$$

where *V*(*p*) is the pixel value of the point *p* within the domain *D* in the 3D volume *V*, *i* and *j* are the pair of pixel values, and *d* is the distance between the pair of points along the direction $\theta$. Then we can obtain fifty matrixes and use their energies, entropies, contrasts, and homogeneities as the 3D GLCM features to enrich the information of the lung nodules.

Finally, the radiomics feature, *FR*, is represented by linking the LBP, HOG, and GLCM features.

### GCN features extraction

CNN can extract effective and robust features adaptively and automatically without dependence on human subjectivity. We utilize the five different architectures of CNN models, AlexNet, GoogLeNet, VGG, ResNet, and AttentionNet for providing a rich and complementary description of lung nodules.

AlexNet is a simple convolutional neural network architecture that consists of five consecutively stacked convolutional layers with a combination of max pooling followed by three fully connected layers. GoogLeNet is a 22-layer deep convolutional neural network that can choose between multiple convolutional filter sizes in each block. VGG has several convolutional layers of $3\times 3$ or $1\times 1$ filters and three fully connected layers. ResNet is a short name for the residual network which can learn the residual function by using the skip connection connecting activations of a layer to further layers. AttentionNet is short for residual attention network which is constructed by stacking multiple attention modules based on the ResNet.

Different models reflect different perspectives on the feature description of nodules. AlexNet tends to capture the important information covering a large area. GoogLeNet holds attention to extract the features at different scales, VGG can generate arbitrarily complex decision features with the help of the non-linear activation functions, ResNet is inclined to solve the problem of vanishing gradient and learn the identity features, and AttentionNet adaptively generates attention-aware features.

Aiming to mine the relationship among these deep features, the GCN is used for learning a function of features in a graph structure, instead of linking these deep features from the different CNN models directly.

A graph consists of a set of nodes and edges, $G= (V, A)$ where node $V_i \in V$ represents the features of the *i*-th node and edge $A_{ij} \in A$ represents the similarity between the features of the *i*-th and *j*-th node. In our work, the nodes are defined by $V = \{ F_{AlexNet}, F_{GoogLeNet}, F_{VGG}, F_{ResNet}, F_{AttentionNet}\}$ where the features are extracted by the five different CNN models. The edges are the adjacency matrix *A*, representing the divergence of different models, which can be defined as

5 $$\begin{aligned} A_{ij}=1-similarity(Net_i,Net_j), \end{aligned}$$

where $similarity(Net_i, Net_j)$ measures the correlation between the ability of *i*-th and *j*-th Net. It is calculated by

6 $$\begin{aligned}{} & {} similarity(Net_i,Net_j)=\sum \limits _{k \in S}\frac{Bf(k)}{S} \nonumber \\{} & {} Bf(k)= \left\{ \begin{array}{ll} 1, &{} C_{Net_i} = C_{Net_j} \\ 0, &{} C_{Net_i} \ne C_{Net_j} \end{array}\right. , \end{aligned}$$

where *S* is the number of training samples. *Bf*(.) is the binary function producing 1 when the model $Net_i$ and $Net_j$ make the same prediction on the same sample in the training data or producing 0 when they make a different prediction.

Based on the above definition, the GCN can fuse the features by following the layer-wise propagation rule

7 $$\begin{aligned} H^{l+1}=\sigma \left(\tilde{D}^{-\frac{1}{2}} \tilde{A}\tilde{D}^{- \frac{1}{2}}H^{(l)}W^{(l)}\right), \end{aligned}$$

where $\tilde{A}$ is the adjacency matrix with added self-connections, which is defined by $\tilde{A}=A+I$, *I* is the identity matrix, $\tilde{D}$ is the degree matrix of $\tilde{A}$. $H^{(l)}$ and $W^{(l)}$ are the output and trainable weight matrix at the *l*-th layer, respectively, and $H^{(0)}$ is the input of the graph convolutional network. In our work, the GCN consists of two layers. $\sigma (.)$ is the activation function, ReLU.

Finally, the fused features by GCN, *FG*, are obtained by concatenating the features of five nodes at the last layer.

### Classification decision

Each CNN model can predict the probability of each nodule belonging to the malignant or benign nodules according to its training schedule. To improve the classification performance, we fine-tune each CNN model by mixing the radiomics features (*FR*), GCN features (*FG*), and its original CNN features on its trained model for obtaining complementary and useful information from the other models. To address class imbalance during training and focus learning on hard misclassified nodules, the Focal Loss is used for training each model, which is formulated as follows

8 $$\begin{aligned} L= \left\{ \begin{array}{ll} {-\alpha (1-y')}^{\gamma }\log {(y')}, &{}y=1\\ -(1-\alpha )y'^{\gamma }\log {(1-y')}, &{}y=0 \end{array}\right. , \end{aligned}$$

where *y* and $y'$ are the true labels and predicted probabilities of nodules, $\gamma$ and $\alpha$ are two hyperparameters, adjusting the rate of hard misclassified nodules and balancing the importance of malignant and benign nodules. $\alpha$ is set to 0.25 and $\gamma$ is set to 2 in our experiments.

The ensemble learning integrates the multiple results from the above models into a consistency function for the final classification decision with weighted averaging. Let *N* be the number of models (five in this work), parameter $w_i$ is the weight parameter, and it can be defined by the proportion of accuracy of all models, like

9 $$\begin{aligned} W_i=\frac{{Acc}_i}{\sum _{j=1}^{N}{Acc}_j} \end{aligned}$$

Since the weight of the single model can evaluate its confidence levels of classification ability, the predictions from the models, $P_{{Net}_i}$, are fused to get the final probability *P* belonging to the malignant or benign nodule using a weighting scheme, by

10 $$\begin{aligned} P=\sum \limits _{i=1}^{N}{W_i\times P_{{Net}_i}} \end{aligned}$$

## Experiments

### Dataset and implementation details

The proposed method was evaluated on the LIDC-IDRI dataset [56], which contained 1018 clinical chest CT scans with lung nodules annotated by four experienced radiologists. The malignancy of each nodule was evaluated using a 5-point scale from benign to malignant. In this study, only the nodules whose diameters are bigger than 3 mm and which were annotated by at least three radiologists are included. We calculated the mean malignancy rating scale of each lung nodule and annotated a nodule whose malignancy rating scale $<=2.75$ as benign, a nodule whose malignancy rating in [2.75, 3.125] as uncertain, and a nodule whose malignancy rating scale $>= 3.125$ as malignant. We obtained a final total of 798 lung nodules, including 313 malignant nodules and 485 benign nodules. In addition, we split these nodules into ten disjoint subsets nearly evenly to conduct 10-fold cross-validation experiments and guarantee that the nodules in different subsets came from different patients to avoid bias in measuring performance.

We normalized the slice thickness of these CT scans to 1 mm, and cut the center of the nodules into the cubes with $56 \times 56 \times 8$, which were reshaped into $128 \times 128 \times 8$. The proposed method was mainly implemented using Python 3.7 in the PyTorch framework. The experimental platform was equipped with an NVIDIA GeForce RTX 3080 Ti with 12 GB of memory. The proposed model was trained using the Adam optimizer and the maximum iteration number of 100. The initial learning rate was set as 0.0001 and decreased by a weight decay of $e^{-4}$.

### Evaluation metrics

To evaluate the proposed method quantitatively, six metrics were used. They are accuracy, sensitivity, specificity, precision, F1 Score, and area under the receiver operator curve (AUC). Let TP, TN, FP, and FN be the number of true positives, true negatives, false positives, and false negatives, respectively. The true positive means that a malignant nodule is correctly classified as malignant. The false negative means that a malignant nodule is incorrectly classified as benign. In the same way, true negative means that a benign nodule is correctly classified as benign, whereas false positive means that a benign nodule is incorrectly classified as malignant. The six metrics are calculated as follows:

1. Accuracy measures the ratio of the number of correctly classified nodules to the number of all nodules, like
  11 $$\begin{aligned} Accuracy = \frac{TP+TN}{TP+TN+FP+FN} \end{aligned}$$
2. Sensitivity measures the proportion of malignant nodules that are identified correctly, and it is also called recall. It is calculated as follows
  12 $$\begin{aligned} Sensitivity(Recall)=\frac{TP}{TP+FN} \end{aligned}$$
3. Specificity measures the proportion of benign nodules that are identified correctly and it is calculated as follows
  13 $$\begin{aligned} Specificity = \frac{TN}{TN+FP} \end{aligned}$$
4. Precision is the fraction of retrieved true positive instances among the retrieved positive instances. It is defined by
  14 $$\begin{aligned} Precision = \frac{TP}{TP+FP} \end{aligned}$$
5. The F1 Score is a statistical analysis of binary classification and it is calculated from the precision and recall as
  15 $$\begin{aligned} F1=2 \times \frac{Precision \times Recall}{Precision+Recall} \end{aligned}$$
6. The AUC is sensitive to an imbalance among the classes. The AUC is the area under the ROC curve which is a graphical plot that illustrates the diagnostic ability of a binary classification method as its discrimination threshold is varied. The AUC is equal to the probability that a classifier will rank a randomly chosen positive instance higher than a randomly chosen negative one. For a predictor *f*, an unbiased estimator of its AUC can be expressed by
  16 $$\begin{aligned} AUC(f) = \frac{\sum _{t_0 \in D^0}\sum _{t_1 \in D^1} {B(f(t_0)<f(t_1))}}{\vert {D^0} \vert \times \vert {D^{1}} \vert }, \end{aligned}$$
  where $B(f(t_0)<f(t_1))$ denotes an indicator function which returns 1 iff $f(t_0)<f(t_1)$, otherwise return 0. $D^0$ is the set of negative samples and $D^1$ is the set of positive samples.

### Classification results

The classification results of the 10-fold cross-validation evaluating the performance of the proposed RGD model are shown in Table 1. In Table 1, we can see that we can achieve a mean accuracy of 93.25% ± 0.021, a sensitivity of 89.22% ± 0.045, a specificity of 95.82% ± 0.032, a precision of 92.46% ± 0.058, F1-score of 0.9114 ± 0.029, and AUC of 0.9629 ± 0.018. Those good and reliable results with small standard deviations show the effectiveness and robustness of the proposed method on lung nodule classification.

**Table 1:** Performance of proposed RGD model in 10-fold cross-validation experiments

Folds	Accuracy	Sensitivity	Specificity	Precision	F1 Score	AUC
1	93.83%	84.38%	100%	100%	0.9153	0.9566
2	95.00%	90.63%	97.92%	96.67%	0.9355	0.9785
3	95.00%	96.77%	93.88%	90.91%	0.9375	0.9921
4	88.61%	87.10%	89.58%	84.38%	0.8571	0.9516
5	90.91%	82.76%	95.83%	82.31%	0.8727	0.9231
6	93.59%	90.00%	95.83%	93.10%	0.9153	0.9701
7	96.20%	90.32%	100%	100%	0.9492	0.9758
8	94.87%	93.55%	95.74%	93.55%	0.9355	0.9629
9	92.31%	83.33%	97.92%	96.15%	0.8929	0.9688
10	92.21%	93.33%	91.49%	87.50%	0.9032	0.9496
Mean	$93.25\% \pm 0.021$	$89.22\% \pm 0.045$	$95.82\% \pm 0.032$	$92.46\% \pm 0.058$	$0.9114 \pm 0.029$	$0.9629 \pm 0.018$

### Ablation study

In this section, we perform ablation studies to further analyze the effectiveness of the fusion of the radiomics, GCN, and CNN features.

We conduct the classification performance on the single CNN model, the fusion of the radiomics and CNN, the fusion of the GCN and CNN, the fusion of the radiomics, GCN, and single CNN, the fusion of the five CNN models, and list their classification accuracies along with our RGD model in the 10-fold cross-validation experiments in Table 2, where the $CNN_1$, $CNN_2$, $CNN_3$, $CNN_4$, and $CNN_5$ represent the AlexNet, VGG13, Resnet34, Attention56, and GoogLeNet, respectively, $CNN_s$ represents the fused DNN model which merges the above five CNN features together, *R* and *G* are short for radiomics and GCN features, respectively, and ‘$+$’ means the fusion. In Table 2, we can see that the performance can be improved by involving the radiomics or GCN features for most CNN models. Especially, embedding GCN features into the CNN models could greatly improve classification performance. The fusion of the radiomics, GCN, and CNN has a significantly increased rate of 3.24%, 2.37%, 2.06%, 3.80%, and 2.53% compared with the original CNN model, respectively. The $CNN_s$ model has higher average accuracy than the single CNN model, which proves that the multiple and different CNN models can extract discriminant features. The $CNN_s + R$ model has a reduction rate of 1.3% compared with our RGD, which demonstrates again our GCN merging in RGD is a better way of fusing features compared with the fully connected DNN merging. In addition, our RGD achieves the highest accuracy by integrating the multiple decisions from the fused CNN models into a consistent decision with ensemble averaging.

**Table 2:** Accuracy of ablation study in 10-fold cross-validation experiments

Model	1	2	3	4	5	6	7	8	9	10	Mean
$CNN_1$	89.71%	92.71%	88.54%	87.36%	87.50%	88.24%	95.83%	85.12%	91.37%	89.67%	89.61%
$CNN_1+R$	84.50%	91.66%	89.58%	87.36%	88.54%	83.48%	90.63%	83.78%	88.99%	89.66%	87.82%
$CNN_1+G$	92.71%	92.71%	93.75%	89.44%	92.71%	91.07%	95.83%	93.45%	94.79%	89.18%	92.56%
$CNN_1+R+G$	92.71%	91.67%	93.75%	89.45%	92.71%	91.07%	95.83%	93.45%	94.79%	89.66%	92.51%
$CNN_2$	91.67%	91.25%	91.25%	88.58%	90.00%	89.46%	91.17%	91.25%	93.75%	89.42%	90.78%
$CNN_2+R$	88.54%	92.50%	91.25%	88.58%	91.25%	92.14%	95.00%	91.25%	91.07%	90.67%	91.23%
$CNN_2+G$	92.71%	92.50%	95.00%	87.42%	90.00%	94.64%	95.00%	92.32%	92.50%	91.63%	92.37%
$CNN_2+R+G$	91.67%	93.75%	96.25%	88.67%	91.25%	92.32%	97.50%	93.75%	92.50%	91.63%	92.93%
$CNN_3$	91.67%	86.25%	91.25%	88.58%	90.00%	89.46%	91.17%	91.25%	93.75%	89.42%	90.28%
$CNN_3+R$	91.67%	90.00%	90.00%	92.33%	90.00%	85.89%	92.50%	88.57%	90.00%	91.92%	90.29%
$CNN_3+G$	91.67%	92.50%	93.75%	86.17%	88.75%	92.14%	95.00%	93.75%	92.50%	90.38%	91.66%
$CNN_3+R+G$	92.71%	92.50%	95.00%	88.67%	90.00%	90.89%	95.00%	92.50%	92.50%	91.63%	92.14%
$CNN_4$	92.86%	89.29%	94.05%	89.29%	85.90%	88.33%	86.25%	87.50%	90.83%	91.25%	89.56%
$CNN_4+R$	89.77%	92.50%	91.25%	88.57%	87.50%	87.50%	90.00%	88.75%	93.75%	92.50%	90.21%
$CNN_4+G$	93.19%	93.75%	95.00%	87.14%	88.75%	93.33%	96.25%	93.75%	93.75%	91.25%	92.62%
$CNN_4+R+G$	93.19%	93.75%	95.00%	87.14%	90.00%	94.58%	97.50%	93.75%	92.50%	92.50%	92.99%
$CNN_5$	89.77%	90.00%	92.50%	91.07%	86.25%	89.58%	92.32%	93.75%	92.08%	90.00%	90.73%
$CNN_5+R$	89.77%	90.00%	95.00%	88.57%	88.57%	91.25%	93.75%	95.00%	91.25%	90.00%	91.32%
$CNN_5+G$	92.05%	92.50%	96.25%	88.57%	88.75%	92.08%	96.25%	95.00%	92.50%	91.25%	92.52%
$CNN_5+R+G$	93.19%	96.25%	95.00%	88.39%	88.75%	93.75%	96.25%	95.00%	92.50%	91.25%	93.03%
$CNN_s$	93.19%	92.50%	90.00%	87.14%	88.75%	92.50%	93.75%	93.75%	90.00%	95.00%	91.66%
$CNN_s+R$	93.19%	92.5%	90.00%	87.14%	90.00%	93.75%	95.00%	93.75%	91.25%	93.75%	92.03%
*RGD*	93.83%	95.00%	95.00%	88.61%	90.91%	93.59%	96.20%	94.87%	92.31%	92.21%	93.25%

**Fig. 2:**
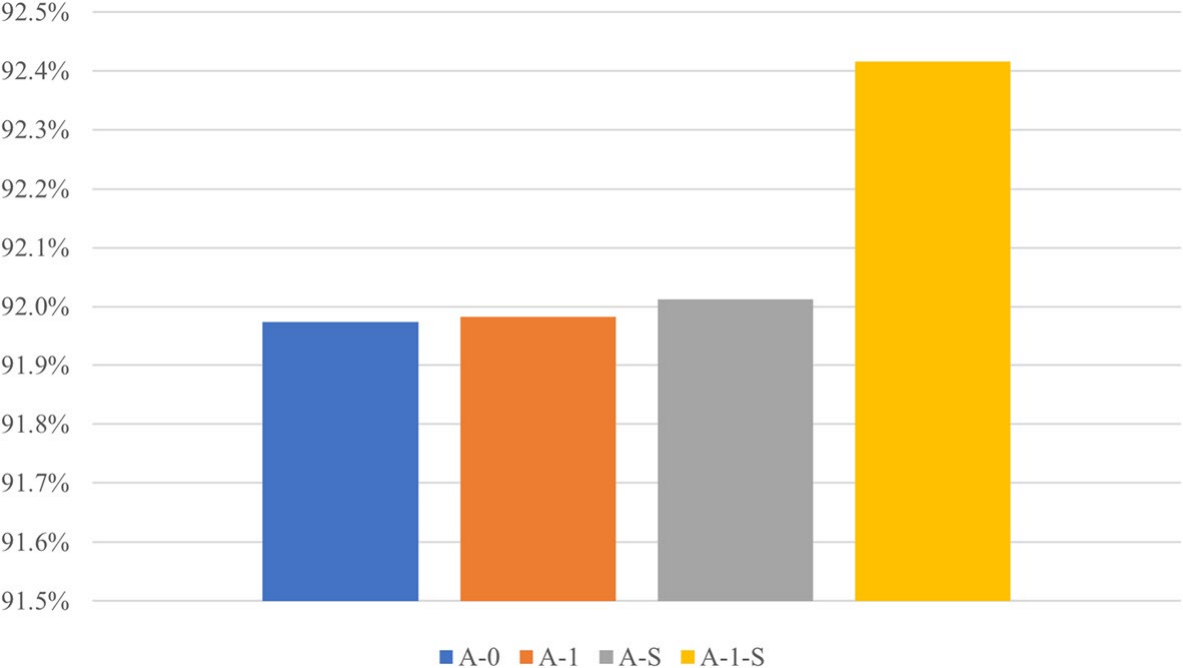
Accuracy of ablation study with the different adjacency matrices in GCN

Then, we test the effect of different adjacency matrices in GCN. We record the effect of different weight functions for the edges in the graph and show the accuracy in Fig. 2. The adjacency matrix A defines the relationship of nodes in the graph. “A-0” is a (0,1)-matrix in which the entries outside the main diagonal are all zero. “A-1” represents a complete graph whose adjacency matrix is an all-ones matrix. “A-S” represents a weight matrix, measuring the similarity of nodes, while “A-1-S” means our proposed adjacency matrix representing the divergence of nodes. In Fig. 2, we can see that any GCN with different adjacency matrices can achieve the above accuracy of 91.9%, which outperforms the single CNN model shown in Table 2. Those results prove the effectiveness of GCN again. In addition, our proposed adjacency matrix measuring the divergence of different models can obtain the highest accuracy, which demonstrates that our method can make full use of important information from the different models and fuse them better for obtaining the discriminative features.

Next, we test the effect of different numbers of CNN models and record the classification performance in Table 3, where *N* is the number of involved CNN models. When *N* is four, we only involve the AlexNet, VGG, ResNet, and AttentionNet models. The ShuffleNet [57] is added to the five current models if *N* is six, while the Mobilenet [58] is added again if *N* is seven. In Table 3, we can see that the classification performance is getting better with the increase of *N* until *N* is six. The performance with *N* of six and seven is worse than that when *N* is four. It indicates that our model with $N = 5$ has reached the best result. In addition, the model has more parameters with the bigger *N*, but the difference is small and almost negligible. The reasoning time is almost the same, no matter what *N* is. Hence, the number of involved CNN models has a certain influence on the effectiveness and does not have much effect on the efficiency. The experiment shows that our proposed RGD model with $N=5$ has the best classification performance.

**Table 3:** Evaluation with different numbers of involved CNN models

N	Accuracy	Sensitivity	Specificity	Precision	F1 Score	AUC	Size	Time
4	92.49%	87.94%	95.39%	92.70%	0.9016	0.9559	14.897743M	1.7ms
5	93.25%	89.22%	95.82%	92.46%	0.9114	0.9629	14.897747M	1.7ms
6	92.31%	87.10%	95.74%	93.10%	0.9000	0.9499	14.897751M	1.7ms
7	92.12%	87.95%	94.77%	91.85%	0.8971	0.9478	14.897755M	1.7ms

**Fig. 3:**
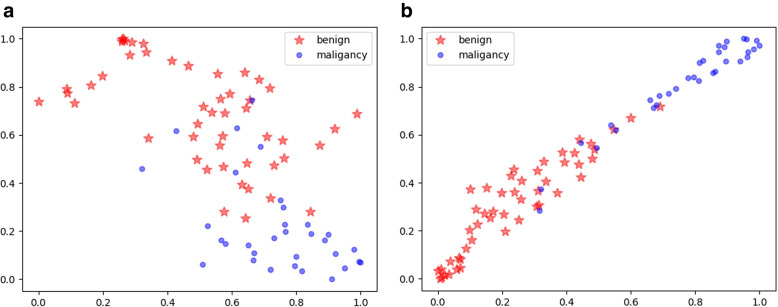
t-SNE embedding using CNN features and the GCN-CNN features. The fused GCN-CNN features can present better differentiation of benign and malignant nodules compared with the CNN features. **a** t-SNE embedding of CNN feature. **b** t-SNE embedding of GCN-CNN feature

Finally, we visualize the CNN features and GCN-embedded CNN features of test data using t-distributed stochastic neighborhood embedding (t-SNE) in Fig. 3, where the benign and malignant nodules are presented by red pentagons and blue circles, respectively. In Fig. 3, we can see that the GCN-CNN features are more compact within classes and enforce a larger angular margin between classes compared with the CNN features. It can be concluded that the aggregation of the GCN feature can enhance feature discrimination and is beneficial to separating the benign and malignant nodules into two distinct groups.

### Comparison with other methods

**Fig. 4:**
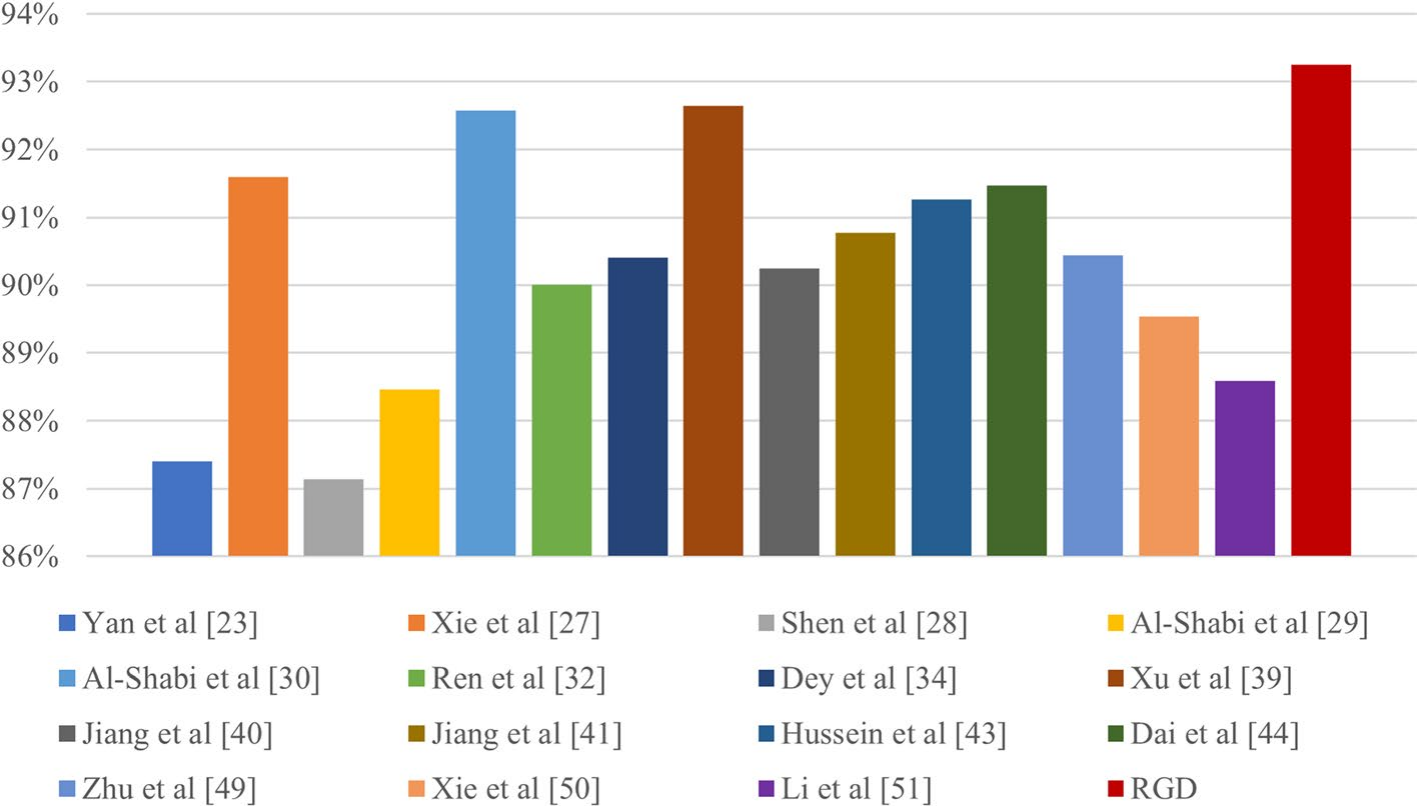
The classification accuracy of the proposed RGD model and the state-of-the-art methods on the LIDC-IDRI dataset

To further evaluate the performance of the proposed RGD, we compared our approach with the state-of-the-art methods of lung nodules in the public LIDC-IDRI database, and the compared results are shown in Fig. 4. Specifically, on the one hand, compared with these methods [23, 27–30, 32], which designed a novel single CNN model by involving the three-dimensional space, multi-scale, multi-view, local and global, and other information for the lung nodule classification, our method outperforms those single-model methods because our model is a mixture model combining the advantages of the different modeling strategies. On the other hand, compared with the multi-model fusion frameworks [34, 39–41], multi-task fusion frameworks [43, 44], and multi-features fusion frameworks [49–51], our model still achieves the highest classification accuracy because the radiomics can capture the relationships between CT image characteristics and nodule classes, graph learning can explore the potential relationships between different deep features to achieve discriminative features, and our model involves them into the five different CNN models training in ensemble schemes for robust performance. The comparison between our RGD model and state-of-the-art methods demonstrates the effectiveness of the proposed method in classifying malignant from benign nodules on this database.

## Discussion

In this study, by fusing the Radiomics and GCN features into five different Deep CNN networks to improve the feature representation, a benign-malignant lung nodule on CT image classification algorithm, named RGD, is established. The extensive experimental results indicated that the proposed model demonstrated outstanding performance in differentiating malignant and benign pulmonary nodules on CT images.

Many automated lung nodule classification approaches have been proposed in the literature and proven to be effective. The deep convolutional neural networks are now increasingly being employed to learn highly representative, hierarchical image features. Multiple CNN models are combined at the feature or decision level to form a new feature representation or learn with optimized decision-making [34, 39–41]. Radiomics signatures have the advantage of differentiating benign and malignant nodules [59, 60]. The fusion of the radiomics and CNN models has been proven to be an efficient strategy [61–63].

In our lung nodule classification method, the radiomics, the graph convolutional network, and five different CNN networks are fused for the first time. Our method can learn the discriminative features between the different CNN features, based on the effective design of an adjacency matrix in a graph convolutional network. With the addition of radiomics, the performance is steadily and robustly improved. Hence, our fused method can effectively separate malignant from benign nodules when tested on the LIDC-IDRI dataset. Sufficient experimental results demonstrate that our method outperforms other fused methods [43, 44, 49–51].

There were some limitations in the current study. On the one hand, radiomics features are sensitive to nodule variability. As shown in Table 2, the model involving the radiomics cannot improve the performance in some folds for some CNN models. On the other hand, there may be overfitting problems for the GCN. In Table 3, we find that the number of nodes in GCN is larger and the performance is worse when *N* is bigger than five.

In the future, we will combine the radiomics in a more complex way, such as fusing the radiomics in front of the network or involving more radiomics features to extract more valuable and robust information about nodules. Moreover, we will integrate the attention module into the graph convolutional network to extract the significant features for reducing the overfitting problem.

## Conclusion

In this study, we propose a fusion algorithm combing the Radiomics, and GCN features into the multiple Deep CNNs, named RGD, for benign-malignant lung nodule classification on CT images. We extract the radiomics features of the pulmonary nodule to characterize the nodule phenotype, capture deep complementary features by using five different CNN models, and utilize the GCN to aggregate optimal features in non-Euclidean space. Finally, we fuse the radiomics features, and the GCN features into the 3D CNNs for generating multiple predictions and acquiring the classification result by ensemble decision. Experimental results demonstrate the effectiveness of the proposed method in extensive ablation studies and comparisons with the current state-of-the-art approaches on the LIDC-IDRI dataset. In the future, we will improve the proposed method and apply it to aid radiologists in the early lung cancer diagnosis process.

## Data Availability

The dataset analyzed during the current study is a public dataset named LIDC-IDRI and is available at https://wiki.cancerimagingarchive.net/pages/viewpage.action?pageId=1966254.
